# Correlates of life course physical activity in participants of the Baltimore longitudinal study of aging

**DOI:** 10.1111/acel.14078

**Published:** 2024-01-16

**Authors:** Ann Zenobia Moore, Eleanor M. Simonsick, Bennett Landman, Jennifer Schrack, Amal A. Wanigatunga, Luigi Ferrucci

**Affiliations:** ^1^ Translational Gerontology Branch, Intramural Research Program National Institute on Aging Baltimore Maryland USA; ^2^ Department of Electrical and Computer Engineering Vanderbilt University Nashville Tennessee USA; ^3^ Department of Epidemiology Johns Hopkins Bloomberg School of Public Health Baltimore Maryland USA; ^4^ Center on Aging and Health Johns Hopkins University Baltimore Maryland USA

**Keywords:** body composition, exercise, life course, physical activity, physical performance

## Abstract

Physical activity is consistently associated with better health and longer life spans. However, the extent to which length and intensity of exercise across the life course impact health outcomes relative to current activity is undefined. Participants of the Baltimore Longitudinal Study of Aging were asked to categorize their level of physical activity in each decade of life from adolescence to the current decade. In linear mixed effects models, self‐reported past levels of physical activity were significantly associated with activity assessed at study visits in the corresponding decade of life either by questionnaire or accelerometry. A pattern of life course physical activity (LCPA) derived by ranking participants on reported activity intensity across multiple decades was consistent with the trajectories of activity estimated from standard physical activity questionnaires assessed at prior study visits. In multivariable linear regression models LCPA was associated with clinical characteristics, measures of body composition and indicators of physical performance independent of current physical activity. After adjustment for minutes of high intensity exercise, LCPA remained significantly associated with peak VO_2_, fasting glucose, thigh muscle area and density, abdominal subcutaneous fat, usual gait speed, lower extremity performance, and multimorbidity (all *p* < 0.01) at the index visit. The observed associations suggest that an estimate of physical activity across decades provides complementary information to information on current activity and reemphasizes the importance of consistently engaging in physical activity over the life course.

## Graphical abstract

In participants of the Baltimore Longitudinal Study of Aging a pattern of life course physical activity (LCPA) derived from self‐reported past levels of activity across multiple decades was associated with clinical characteristics, measures of body composition and indicators of physical performance independent of current physical activity. The observed associations suggest that an estimate of physical activity across decades provides complementary information to measures of current activity.

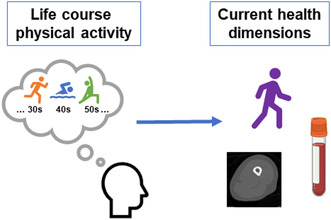

**Abbreviations**
BLSA: Baltimore Longitudinal Study of Aging
CT: computed tomography
FEV1/FVC: forced expiratory volume in the first second/forced vital capacity
HDL: high‐density lipoprotein cholesterol
Health ABC PPB: Health ABC Physical Performance Battery
kcal: kilocalorie
LCPA: life course physical activity
MET: metabolic equivlent of task
RDW: red cell distribution width

## 1 INTRODUCTION

There is evidence that public health interventions that encourage physical activity have the potential to improve the health of individuals and populations and higher physical activity has been consistently associated with better health and longer life spans (Arem et al., 2015; Bull et al., 2020; Lee et al., 2012; Piercy et al., 2018). In studies of older adults, physical activity is associated with better physical performance (Brach et al., 2004; Hall et al., 2017; Spartano et al., 2019; Stenholm et al., 2016), less disability (Ferrucci et al., 1999; Sanchez‐Sanchez et al., 2020; Tak et al., 2013), lower risk of cardiovascular events (Mora et al., 2007; Wahid et al., 2016), improved glucose homeostasis (Boniol et al., 2017; Smith et al., 2016), and lower depressive symptoms (Bridle et al., 2012; Schuch et al., 2018; Zhang et al., 2021) among other outcomes. Evidence suggests that these positive health outcomes are mediated, at least in part, by the enhancement of biological resilience mechanisms that have been described as the hallmarks of aging (Lopez‐Otin et al., 2013) such as epigenetic alterations, genomic instability, mitochondrial dysfunction, and cell senescence (Rebelo‐Marques et al., 2018). For example, physical activity has been associated with second‐generation DNA methylation based epigenetic age measures, PhenoAge and GrimAge (Fox et al., 2023; Kresovich et al., 2021), as well as other forms of epigenetic alteration (Widmann et al., 2019). The relationship between physical activity and mitochondrial biogenesis as well as mitophagy, expansion and enhancement of the mitochondrial network, is well established especially in the context of endurance exercise (Jornayvaz & Shulman, 2010; Picca et al., 2023). Physical activity is also implicated more broadly in cellular maintenance mechanisms (Radak et al., 2019).

While a wealth of studies have tested the beneficial effects of physical activity in model systems, population based studies as well as clinical trials, most published research only considers current physical activity level, without considering the length and intensity of physical activity performed prior to study enrollment. Current physical activity may be a proxy for usual physical activity, but it is plausible that while some individuals have been physically active over their entire lives, for some becoming physically active may have been a more recent decision and for others limited or low activity may be a recent occurrence due to aging‐ and or health‐related limitations. To this end, information on length and intensity of physical activity over the life course may be important to estimate its beneficial effects independent of current physical activity engagement.

Studies of mid‐life physical activity and outcomes in later life demonstrate the potential importance of life course activity, suggesting that exercise at mid‐life may improve muscle characteristics, physical performance, and mobility (Akune et al., 2014; Cooper et al., 2011; Edholm et al., 2019, 2021; Leino‐Arjas et al., 2004; Patel et al., 2006; Stenholm et al., 2016), yet characterizing life course physical activity (LCPA) in population‐based studies remains challenging. Some instruments reference specific periods, such as mid‐life, establishing an age floor for the contribution of participant data, which limits subsequent inferences. Instruments that collect detailed temporal information may require substantial participant effort to complete. Here we describe the implementation of a brief physical activity history questionnaire that was designed to collect information on both current and prior level of physical activity in participants of the Baltimore Longitudinal Study of Aging (BLSA). Building on questions from the Health ABC and InCHIANTI studies (Patel et al., 2006) participants were asked to categorize their level of physical activity in each decade of life from adolescence to the current decade. The structure of the physical activity history questionnaire allowed for the collection of data in participants across the adult age range and provided information on physical activity prior to study enrollment. A pattern of LCPA was derived using information that participants provided on the level of activity over multiple decades. We hypothesized that recalled levels of physical activity for each decade would be correlated with self‐reported and/or measured current physical activity information collected during the corresponding period of life. Further we anticipated that, consistent with the hypothesis that more physical activity across the life course increases reserve and promotes resilience, life course patterns of activity that incorporate more periods during which participants engage in more activity at greater frequency or for longer time periods would be positively associated with current physical function and lean body composition as well as negatively correlated with indicators of subclinical disease, independent of current physical activity.

## 2 METHODS

### 2.1 Study sample

Begun in 1958, the BLSA is the longest running study of human aging in the United States. The study is a continuously enrolled cohort of community dwelling adults age 20+. Study participants are healthy at study entry based on strict eligibility criteria and are followed up according to age dependent intervals. BLSA enrollment and study protocols have been previously described (Kuo et al., 2020). In person study visits are conducted through the Clinical Research Unit of the National Institute on Aging Intramural Research Program and incorporate comprehensive clinical and laboratory testing as well as measures of physical and cognitive performance. During a suspension of regular study visits due to the COVID‐19 pandemic BLSA, participants were invited to take part in questionnaires by phone and mail. The LCPA variable described below is based on data from a physical activity history questionnaire distributed in early 2021 with responses received between 7 January 2021 and 19 May 2021. The study sample for the analyses presented here includes participants with LCPA and data for questionnaire‐based estimates of current physical activity at their most recent in‐person study visit as well as key covariates (*n* = 690).

### 2.2 Life course physical activity

**FIGURE 1:**
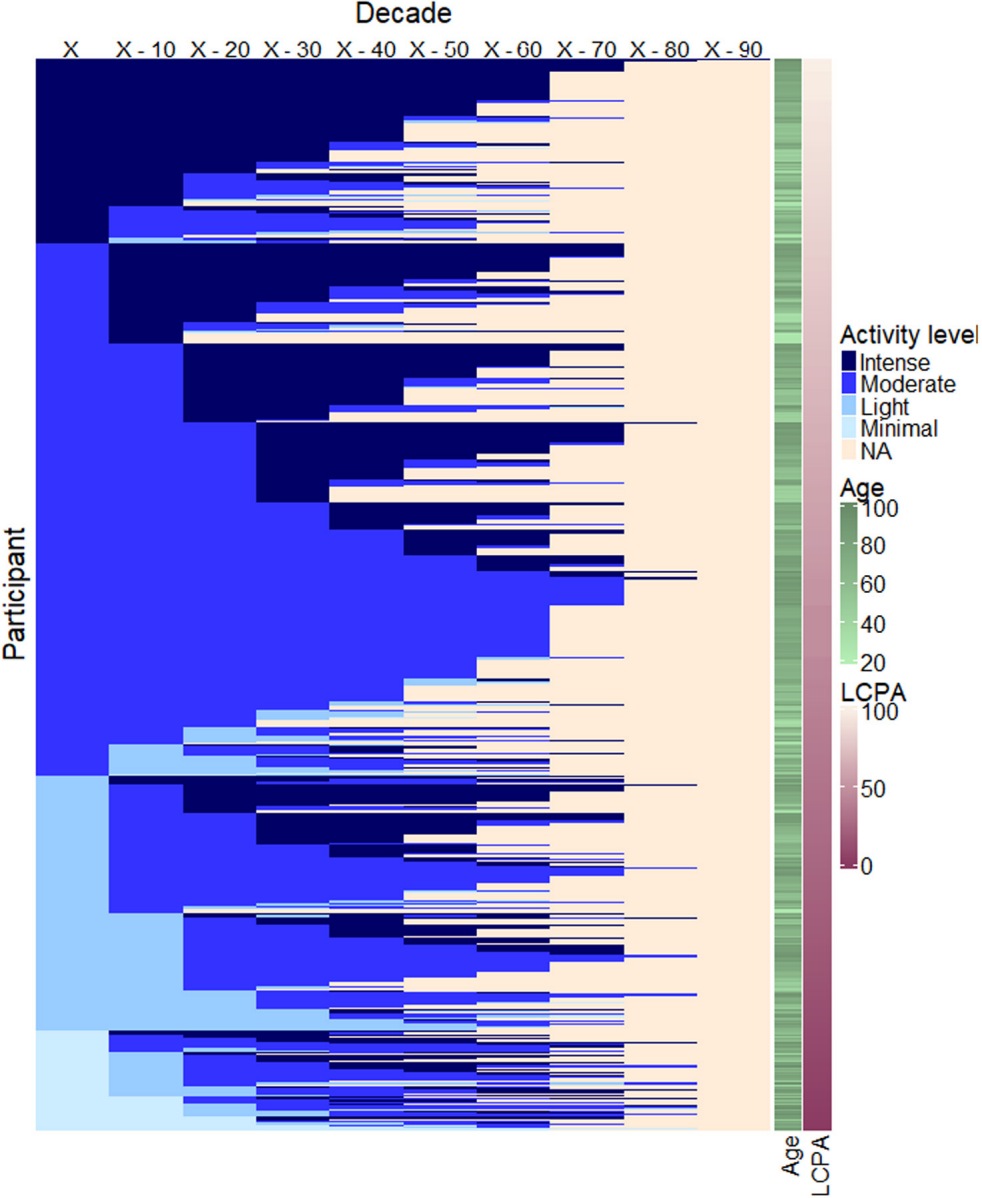
Responses to the physical activity history questionnaire among participants of the BLSA in the study sample (*n* = 690). The columns of the heatmap are ordered by decade where X is the decade of age at the index visit and each column to the right is a preceding decade. Rows are ordered by LCPA value from highest at the top to lowest at the bottom of the figure. BLSA, Baltimore Longitudinal Study of Aging; LCPA, life course physical activity.

Physical activity history was ascertained by questionnaire: participants were asked to categorize their activity level in each decade from adolescence, age 10–19 years old, through their current age as minimal (mostly sitting some walking), light (low intensity exercise 2–4 h/week), moderate (low intensity exercise ≥5 h/week or medium intensity exercise ≥3 h/week) or intense (medium to high intensity exercise ≥5 h/week) (Appendix S1). Because the questionnaire was implemented between study visits, the most recent visit before administration was designated as the index visit. Questionnaire responses corresponding to a decade of life after the index visit were excluded. Participants who did not report an activity level for the index visit decade or who reported history of physical activity for fewer than two decades were excluded. To summarize the pattern of physical activity across decades, the responses for each participant were reversed so that each individual was represented by a sequence of activity levels starting with the current decade and ending with their response for adolescence. These sequences were ordered based on activity level in the current decade and then each preceding decade (Figure 1); to generate LCPA, participants were ranked based on this order and the rank was scaled to 100.

### 2.3 Questionnaire based current physical activity

Physical activity has been captured in the BLSA since 2004 using an instrument initially developed for the Health ABC study (Brach et al., 2004) based on the Minnesota Leisure Time Physical Activity Questionnaire (Taylor et al., 1978). Metabolic equivalent of task (MET) estimates are derived from participant self‐report of the amount of time spent engaging in a range of household, exercise, and recreational activities in the past 2 weeks (Ainsworth et al., 1993). A range of summary variables including total kilocalorie (kcal) per week and minutes of high intensity exercise per week are derived from the questionnaire data. One index visit total kcal value was a clear outlier (>200,000 kcal/week) and led to the exclusion of the participant from the analytical sample.

### 2.4 Accelerometer based physical activity

A wrist‐worn accelerometer (Actigraph GT9X monitor, Actigraph, Pensacola, FL) has been used to capture objective measurements of free‐living physical activity in the BLSA since 2015. The accelerometer was fit during the last day of clinic visits and participants wore the monitor continuously for up to 7 consecutive days (Wanigatunga et al., 2021). Data were downloaded and processed using the ActiLife software (v 6.13.4). Participants who collected fewer than 3 valid days of data (valid day defined as <10% missing data) were excluded. Active minutes per day and total activity count per day were quantified as previously described using the ARC Tools package (Karas et al., 2021; Schrack et al., 2019; Wanigatunga et al., 2021).

### 2.5 Body composition

Computed tomography (CT) scans including images of the abdomen (L4‐L5) and thigh (mid‐femur) are acquired by the Radiology Department of MedStar Harbor Hospital (Baltimore, MD) using a Somatom Sensation 10 CT scanner (Siemens, Malvern, PA). Tissue types and regions are segmented and quantified using customized machine learning algorithms specific to each body region with manual review and correction as appropriate (Yang et al., 2022; Yu et al., 2022). Variables of interest include areas of abdominal subcutaneous and visceral fat, thigh subcutaneous and inter‐muscular fat areas, as well as thigh muscle area (mm^2^), and thigh muscle intensity, Hounsfield unit (HU), an indicator of lipid content (Goodpaster et al., 2000).

### 2.6 Demographic and health behaviors

Demographic and health behaviors, age, sex, race, education, and smoking status, are ascertained using a structured interview. In the analyses described here self‐identified race is represented by a binary variable (Black or African‐American versus all other groups), education is categorized as highest grade in school completed equal to less than college, college graduate or post college, and smoking status is represented by three categories (never smoker/former smoker/current smoker).

### 2.7 Other clinical measurements

Blood for clinical laboratory measurements is drawn after an overnight fast and assays are performed in the Clinical Laboratory Improvement Amendments (CLIA) certified clinical laboratory of MedStar Harbor Hospital (Baltimore, Maryland). High‐density lipoprotein cholesterol (HDL), and triglycerides are measured using an Atellica analyzer (Siemens). Hemoglobin and red cell distribution width (RDW) are measured using a SYSMEX SE‐2100 (Sysmex, Kobe, Japan). Fasting glucose and fasting insulin are measured as part of an oral glucose tolerance test: after a 10‐h overnight fast, participants consume a 75‐g glucose solution blood samples are collected just before glucose ingestion and every 20 min for 120 min.

Height (cm) as well as other clinical and functional measurements are objectively assessed using standardized protocols by trained clinical and research study staff. Peak VO_2_ (mL/kg/min) is measured during a treadmill test using a modified Balke protocol (Fleg et al., 2005). Indicators of pulmonary function were measured using a MedGraphics Gas Exchange System (Medical Graphics Corp, St Paul, MN) through January 2020 currently a Vyaire Medical Vyntus CPX (Vyaire Medical, Inc. Mettawa, IL) is used and the ratio of forced expiratory volume in the first second to forced vital capacity (FEV1/FVC) was calculated. Usual gait speed (m/s) is measured over a 6‐m course and is a component of the Health ABC Physical Performance Battery (Health ABC PPB) (Simonsick et al., 2001) which also includes assessment of repeated chair stands, standing balance and a narrow walk.

### 2.8 Multimorbidity

Multimorbidity was defined by the sum of positive observations across indicators for 15 conditions: hip fracture, cancer other than basal cell cancer, anemia, hypertension, diabetes, coronary artery disease, depressive symptoms, peripheral artery disease, chronic kidney disease, chronic obstructive pulmonary disease, congestive heart failure, lower extremity joint pain, stroke, Parkinson's disease, and cognitive impairment (Fabbri et al., 2015). Disease indicators were defined based upon combinations of self‐report, clinical and laboratory measures, as well as medication use. Multimorbidity was defined as missing if information was missing for more than three conditions.

### 2.9 Statistical analyses

Descriptive statistics were used to characterize the distribution of demographic characteristics, health behaviors and technical covariates by LCPA in the study sample.

To understand the impact of recalling activity over longer periods of time, the relationships between responses to individual questions from the physical activity history questionnaire and assessments of current activity at the time of the study visits were evaluated. The associations between questionnaire based and accelerometer based physical activity metrics at the index visit and activity level reported in the corresponding age decade were described graphically and in unadjusted linear regression models. Subsequently all available observations of questionnaire and accelerometer data were evaluated in longitudinal models: coefficients from linear mixed effects models with random intercept for each questionnaire based (total kcal, minutes of high intensity exercise) and accelerometer based (total activity count/100,000 and active minutes) concurrent physical activity metric were evaluated, models included indicator variables for recalled activity level, years prior to the index visit was the time axis for analysis and interaction terms between activity level and time were also included.

To discern whether LCPA, the summary measure derived from responses to the physical activity history questionnaire, corresponds to true individual trajectories of physical activity over time, LCPA was compared to trajectories of accelerometer based and questionnaire based current activity estimated at the time of study visits. Individual slopes were estimated for participants with at least two observations of the current activity measure in linear mixed effect models with random intercept and random slope, using the time axis, years prior to the index visit, as the independent variable. Estimates were obtained from models for total kcal, ln(minutes high intensity exercise +1), total activity count/100,000, and active minutes. The association between LCPA and each slope variable was estimated in a simple linear regression model.

Further analyses were aimed to determine whether the correlations of selected quantitative clinical and functional characteristics with LCPA were similar to the correlations of these same measures with trajectories of physical activity over time directly assessed using accelerometry or administered questionnaires over multiple follow‐up visits. Specifically, we compared the magnitude and direction of coefficients from multivariable linear regression models including LCPA to models including slopes (described above) derived from the two types of assessment.

All models were adjusted for age at the index visit centered at 70 years, sex, race, education, smoking status, and phone versus mailed questionnaire. Quantitative characteristics of interest, physical activity variables, and LCPA were standardized for analysis. Models for peak VO_2_, body composition measures and physical performance measures were further adjusted for height centered at 170 cm.

The association between LCPA and selected quantitative characteristics independent of current physical activity was estimated using multivariable linear regression models. The covariates for these models were the same as those used for the comparison with physical activity trajectories. Standardized variables were also used in these models. Models were estimated for all dependent variables and LCPA with adjustment for each measure of current physical activity separately: four models for each dependent variable.

All analyses were conducted in R version 4.2.0 (R Core Team, 2022), the ComplexHeatmap package (Gu et al., 2016) was used for Figure 1.

## 3 RESULTS

The study sample includes 690 BLSA participants, mean age 68 years (range: 22–102 years) and 43.9% male (Table 1). One hundred seventy‐four (25.2%) participants identify as Black, 480 participants as White (68.1%), and 46 participants self‐identify with other groups including Asian, Pacific Islander, American Indian or Alaska Native, and Other. Overall, 332 different response patterns were observed among the answers to the physical activity history questionnaire (Figure 1). The most common pattern (*n* = 33, 4.8%) was a report of moderate activity in every decade of life for a participant in their 70s at the index visit. LCPA was higher among younger participants, never smokers and participants with higher education as well as participants who answered the physical activity history questionnaire by phone (Table 1).

**TABLE 1:** Demographic characteristic and distribution of LCPA at the index visit among BLSA participants in the study sample (*n* = 690).

Characteristic	*n* (%)	LCPA, mean (sd)	*p*‐value*
Age < 60	195 (28.3)	54.2 (28.2)	<0.001
Age 60– 80	338 (49.0)	50.9 (28.4)	
Age > 80	157 (22.8)	43.2 (29.7)	
Female	387 (56.1)	50.4 (29.2)	0.721
Male	303 (43.9)	49.6 (28.5)	
Non‐black	516 (74.8)	50.9 (28.7)	0.186
Black	174 (25.2)	47.5 (29.4)	
<College graduate	87 (12.6)	44.9 (31.5)	0.070
College graduate	155 (22.5)	49.7 (28.2)	
Post college	448 (64.9)	51.2 (28.5)	
Never smoker	475 (68.8)	51.5 (29.2)	0.027
Former	188 (27.2)	47.7 (27.6)	
Current	27 (3.9)	41.0 (30.0)	
Phone interview	538 (78.0)	51.4 (29.2)	0.016
Mail interview	152 (22.0)	45.3 (27.2)	
Time lag <2 years^a^	283 (41.0)	50.0 (28.9)	0.925
Time lag 2–4 years^a^	240 (34.8)	50.5 (29.5)	
Time lag >4 years^a^	167 (24.2)	49.6 (28.1)	

Abbreviations: BLSA, Baltimore Longitudinal Study of Aging; LCPA, life course physical activity.

^a^ Years between physical activity history questionnaire and index study visit.

* ANOVA *p*‐value for 3 level variables, and *t*‐test *p*‐value for 2 level variables.

### 3.1 Correlation between individual activity estimates retrospectively and current activity assessed at the time of the visit

**FIGURE 2:**
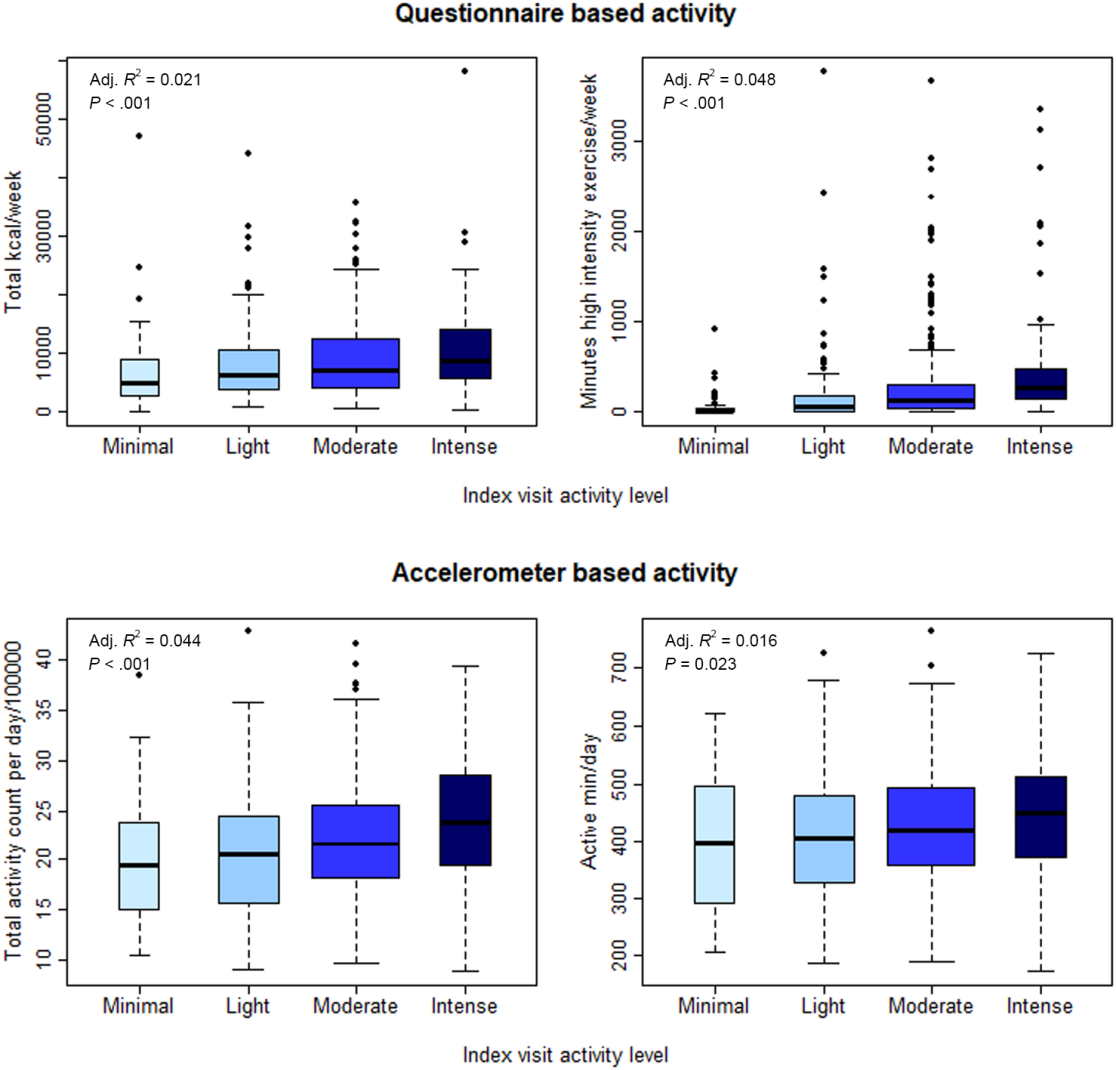
Distribution of questionnaire based (total kilocalorie/week, minutes high intensity exercise/week), and accelerometer based (total activity count per day/100,000, active min/day) physical activity at the index visit by activity level in the corresponding decade of the physical activity history questionnaire (*p*‐values from ANOVA).

Levels of activity reported on the physical activity history questionnaire were associated with both questionnaire‐based and accelerometer‐based current physical activity assessed at the index visit (Figure 2; Table S1). In unadjusted linear regression models, participants reporting moderate and intense activity in the decade corresponding to the index visit reported significantly more minutes of high intensity exercise per week by questionnaire at the index visit (respectively *p* = 0.001 and <0.001). Specifically, model estimates suggest that participants who reported minimal activity had less than an hour of high intensity exercise per week while participants indicating an intense activity level had greater than 7 h per week at the index visit (*β*_intercept_ (se) = 53 (55), *β*_Intense_ (se) = 377 (69)). To evaluate the association between individual physical activity history questionnaire responses and all available activity observations, activity levels in each decade were evaluated in mixed effects models with random intercept incorporating up to 16.3 years of questionnaire based physical activity observations or 5.0 years of accelerometer observations. Reported moderate or intense activity level in a decade was positively associated with all concurrently ascertained activity measures (Table S2). For example, the model estimating accelerometer based active min/day suggests that participants who reported moderate activity had approximately half an hour more active time than participants reporting minimal activity (*β*_moderate_ (se) = 33.4 (14.8)) However, a significant interaction between activity level and the time between assessments suggests that the reliability of recalled activity levels was attenuated over time, e.g., the estimated difference in concurrently measured active time for participants reporting minimal and moderate activity is approximately nine minutes smaller with each year prior to the index visit (*β*_moderate×years prior_ (se) = −9.3 (4.7)) (Table S2).

### 3.2 Correlation between LCPA and physical activity trajectory

**FIGURE 3:**
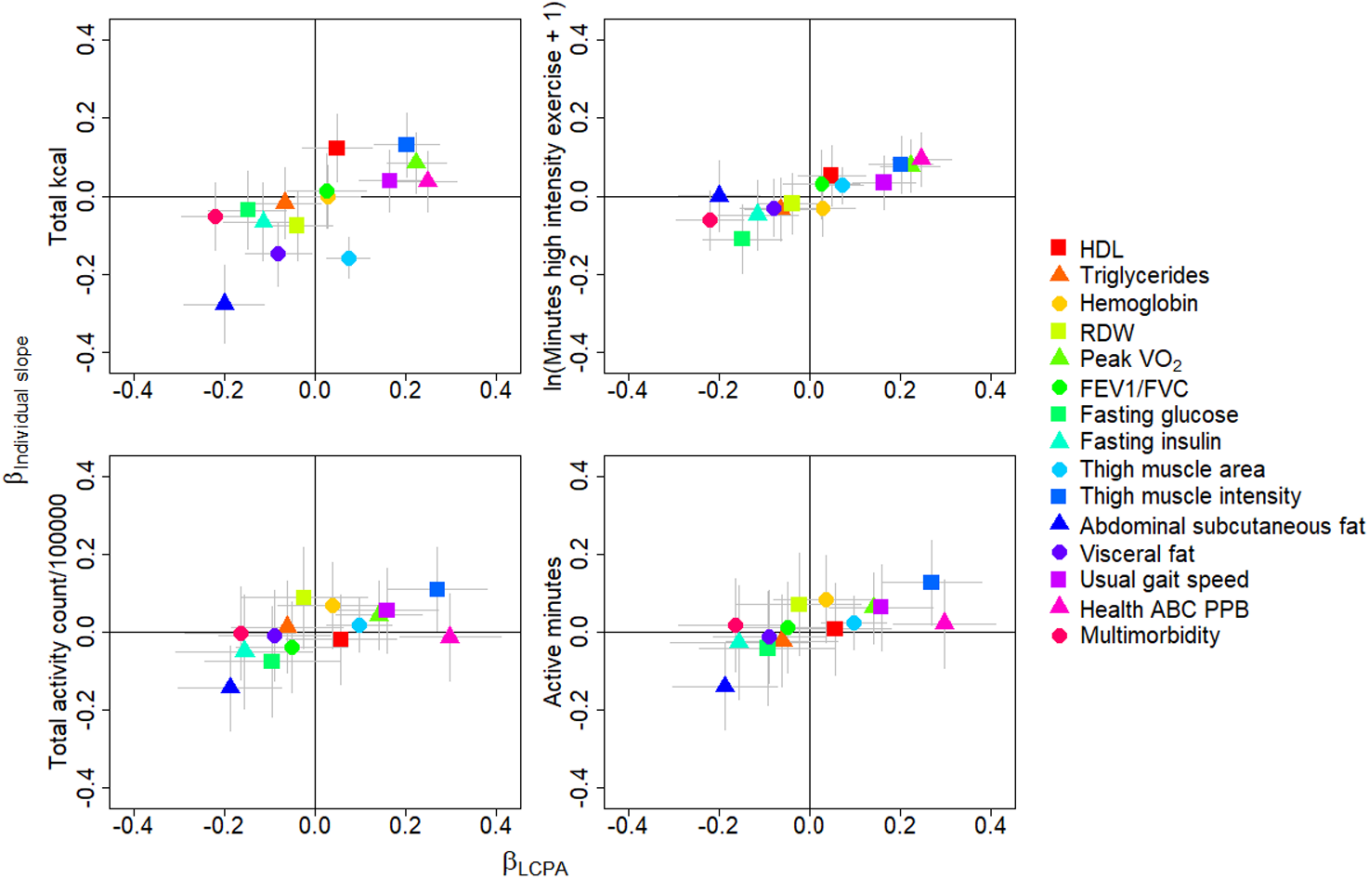
Comparison of coefficients estimating the association between quantitative characteristics and scaled life course physical activity and scaled questionnaire based or accelerometer based physical activity slope from separate multivariable linear regression models adjusted for age at the index visit, sex, race (non‐black/black), education, smoking status, and phone versus mailed questionnaire. Models for peak VO_2_, body composition, and physical performance were also adjusted for height.

In linear regression models, LCPA appears consistent with the trajectory of physical activity over time: while LCPA was not associated with individual time slope estimates for total kcal, it was positively associated with individual time slope estimates for minutes of high intensity exercise per week, total activity count per day and active minutes per day: *p*‐value <0.01 for all three analyses (Figure S1; Table S3). To evaluate whether LCPA and physical activity trajectory had similar relationships to selected clinical characteristics, coefficients from separate multivariable linear regression models for measures of clinical characteristics, body composition and physical performance (assessed at the index visit) were qualitatively compared: coefficients for LCPA were mostly similar in magnitude and direction to the effect estimates for physical activity trajectory as estimated by individual slopes (Figure 3; Table S4). Interestingly, after adjustment for demographic and technical covariates, LCPA was significantly associated with some characteristics of interest while the corresponding model using current physical activity evaluated at each visit was not. For example, when standardized usual gait speed was the dependent variable the coefficient for standardized LCPA was 0.17 (*p* < 0.001) while in the comparable model the coefficient for standardized total kcal was 0.04 (*p* = 0.331) (Table S4).

### 3.3 LCPA and clinical characteristics, body composition, and physical performance

**FIGURE 4:**
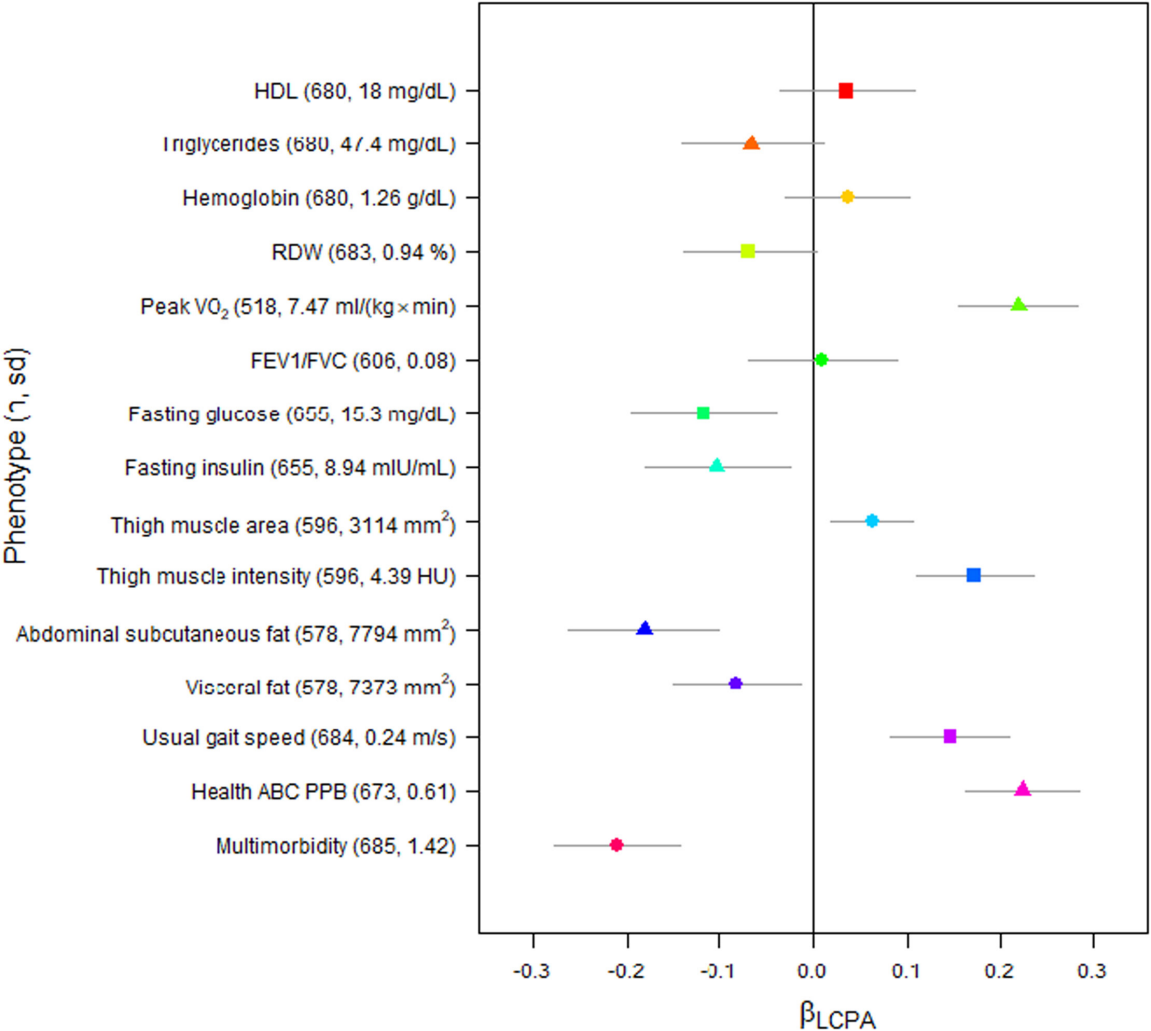
Coefficients from multivariable linear regression models estimating the association between standardized LCPA and standardized quantitative characteristics adjusted for minutes high intensity exercise as well as demographic and technical covariates. LCPA, life course physical activity.

The association of LCPA with characteristics of interest adjusted for current physical activity was evaluated in multivariable linear regression models. Higher LCPA was consistent with higher HDL, hemoglobin, peak VO_2_, FEV1/FVC, thigh muscle area, thigh muscle intensity, usual gait speed and Health ABC PPB, while triglycerides, RDW, fasting glucose, fasting insulin, abdominal subcutaneous fat, visceral fat, and multimorbidity were all lower (Figure 4; Table S5). After adjustment for minutes of high intensity exercise at the index visit, LCPA was still significantly associated (*p* < 0.05) with peak VO_2_, fasting glucose, fasting insulin, all measures of body composition, physical performance, as well as multimorbidity in the direction expected under the assumption that LCPA conveys a beneficial effect (Figure 4). Among participants a standard deviation higher LCPA was associated with an estimated difference of 1.95 cm^2^ (*p* = 0.005) in thigh muscle area and 0.03 m/s (*p* < 0.001) difference in gait speed (Table S5). In these models, minutes of high intensity exercise at the index visit was associated with HDL, peak VO_2_, measures of body composition except thigh muscle area and usual gait speed (Table S5). Results were similar when models were adjusted for total kcal, minutes of high intensity exercise, total activity count, or active minutes (Table S5). In most cases, LCPA was associated with several health‐related characteristics adjusted for current physical activity.

## 4 DISCUSSION

We used a brief physical activity history questionnaire to summarize the pattern of physical activity amount and intensity across the life course and derived a measure of LCPA. The summary measure, LCPA, was based on the rank of participants when they were ordered by activity level reported in the current decade of life followed by earlier periods. Using this algorithm to define LCPA, the most recent physical activity was prioritized rather than specific age ranges such as mid‐life or adolescence: participants could be scored regardless of age. Between participants with similar questionnaire response patterns, those with more decades of information were assigned higher ranks and better values of LCPA. Despite this preference for longer histories, on average older participants had lower values of LCPA. The questionnaire and derived variable demonstrated a diversity in histories of physical activity across BLSA participants that is not exclusively related to or limited by age.

Self‐reported history of physical activity was positively correlated with other measures of physical activity at the index visit including both questionnaire‐based and accelerometer‐based metrics in a dose response manner. The ranges of concurrently measured physical activity corresponding to minimal, light, moderate, and intense activity level responses were overlapping and the relationship with other measures attenuated when comparing responses from earlier decades to activity measured at prior visits suggesting greater misclassification for periods of life that were farther back in time. Misclassification of individual responses may compound in downstream summary measures such as LCPA and diminish observed associations with phenotypes. However, the consistent positive association, with physical activity measures at the index visit as well as in the evaluation of activity information from all available study visits suggests that the individual questionnaire items do capture broad trends in physical activity and may be useful for assessing the effects of habitual physical activity over the life course, rather than just late life.

LCPA was moderately associated with the estimated trajectory of questionnaire‐based minutes of high intensity exercise per week as well as both accelerometer‐based physical activity measures in BLSA participants and the associations between LCPA and a range of health‐related outcomes were overwhelmingly similar to the associations of these outcomes with trajectories of all tested measures of physical activity that were derived from assessments at study visits. The correlation between these two different methods of capturing the pattern of physical activity over time suggests that LCPA, the derived pattern variable based on data collection at a single time point, may reflect some of the same information as data collected over time through individual, serial visits. While the BLSA does follow participants longitudinally, adults may join at any age (20 years or older) and many participants join the study in late middle age or as older adults: data on earlier life exposures is largely absent. This gap is not unique to the BLSA and even limited information on prior exposure may be useful in understanding differences in health outcomes across otherwise similar participants.

Finally, in models consistent with a cross‐sectional study of BLSA data we observed that, independent of current physical activity, higher LCPA was associated with several clinical and functional measures in a manner consistent with the assumption that LCPA conveys beneficial effects of physical activity compounded over time: higher muscle mass and density in CT imaging at the thigh, less fat across the abdomen, better lower extremity performance, and higher cardiorespiratory fitness. Changes in visceral fat have been observed with physical activity interventions (Armstrong et al., 2022) and our observations are consistent with cross‐sectional evaluation of abdominal fat measurements in the CARDIA study (Whitaker et al., 2017). Our observations on fitness are consistent with Nayor and colleagues who show that higher accelerometer based physical activity measured at two time points approximately 8 years apart were associated with higher peak cardiorespiratory fitness relative to other combinations of low and high activity among participants of the Framingham Heart Study (Nayor et al., 2021). While the direction of association for all clinical laboratory measures was consistent with the direction anticipated for better health outcomes, the absence of statistically significant associations with HDL, triglycerides and hemoglobin may reflect the importance of other factors such as current or life course diet. Negative associations with fasting glucose and fasting insulin are consistent with the observed benefits of exercise on glucose metabolism (Boniol et al., 2017; Smith et al., 2016). Association with lower multimorbidity suggests that activity across the life course may reduce the prevalence of geriatric syndromes. This association echoes observations in the National Institutes of Health‐AARP Diet and Health Study where patterns of maintaining or increasing leisure‐time physical activity based upon recall of activity during periods up to age 61 were associated with lower mortality (Saint‐Maurice et al., 2019). Since individuals with several chronic conditions are not able to exercise, we cannot exclude that the observed association may be due to reverse causation.

There are several limitations specific to the current evaluation of LCPA. The study sample is relatively small and the BLSA is a unique population that is not representative of the general US adult population: the range and diversity of life course patterns of physical activity is limited. The comparison with concurrently collected physical activity was constrained to comparisons with data collected using current study methods and associations with longer trajectories or data from earlier in the life course may also be informative. The method of capturing and summarizing the pattern of physical activity used here also has limitations. The categories of activity in the physical activity history questionnaire are broad and the wording of examples may not prompt participants to sufficiently consider incidental activity: there is a lack of precision and potential misclassification in individual items. Further the use of a rank in the algorithm used to summarize the pattern of physical activity means that LCPA values would not be directly comparable across populations. We also note a potential drawback in our analytic strategy: it is plausible that some observed associations with outcomes have occurred by chance alone given the number of tests performed to capture a wide range of anticipated relationships.

Despite these limitations we believe use of this questionnaire, definition of the summary physical activity pattern variable, and association of this variable with several phenotypes represents an important proof of concept for capturing a life course exposure prior to study enrollment in studies targeting enrollment of, or sample collection in, middle‐age to older adults. Further, the independent association of LCPA with clinical, body composition and physical performance measures suggest that even broad estimates of volitional physical activity across decades provides complementary information to current activity estimates that may have consequences for future health related outcomes. Our observations reemphasize the importance of consistently engaging in physical activity across the life course.

## AUTHOR CONTRIBUTIONS

AZM, EMS, and LF contributed to the study conception and design. Aspects of data collection and processing were overseen by each author. Analyses were completed by AZM. All authors contributed to and approved the final manuscript.

## CONFLICT OF INTEREST STATEMENT

Jennifer Schrack works as a consultant for Edwards Lifesciences.

## Supporting information


Appendix S1:

## Data Availability

Data from the Baltimore Longitudinal Study of Aging may be requested through the study website: blsa.nih.gov.
