# Supplementary material for: Correlates of life course physical activity in participants of the Baltimore longitudinal study of aging
Source: Aging Cell. 2024 Jan 16;23(4):e14078. doi: 10.1111/acel.14078 (PMC11019133; doi:10.1111/acel.14078)

**Supplementary Figures, Tables, and Text**

Supplementary table 1: Coefficients from simple linear regression models evaluating the association between questionnaire based physical activity as well as accelerometer based physical activity at the index visit and activity level reported on the physical activity history questionnaire in the corresponding decade of life. Minimal activity is the reference category in each model.

|  | Total kilocalorie/week  (n = 690) | | Minutes high intensity exercise/week  (n = 690) | | Total activity count per day/100000  (n = 404) | | Active minutes/day  (n = 404) | |
| --- | --- | --- | --- | --- | --- | --- | --- | --- |
|  | β (se) | p | β (se) | p | β (se) | p | β (se) | p |
| *(Intercept)* | *6642 (835)* | *<.001* | *53 (55)* | *0.340* | *19.90 (0.96)* | *<.001* | *400.2 (16.8)* | *<.001* |
| Light | 1274 (984) | 0.196 | 115 (65) | 0.079 | 0.53 (1.14) | 0.644 | 3.7 (19.9) | 0.851 |
| Moderate | **2224 (909)** | **0.015** | **200 (60)** | **0.001** | **2.28 (1.05)** | **0.031** | 28.3 (18.3) | 0.123 |
| Intense | **3915 (1036)** | **<.001** | **377 (69)** | **<.001** | **4.46 (1.21)** | **<.001** | **48.4 (21.0)** | **0.022** |

Supplementary table 2: Coefficients from linear mixed effects models with random intercept estimating the association between all observations of questionnaire based current physical activity as well as accelerometer based physical activity and activity level in the corresponding decades as reported on the physical activity history questionnaire.

|  | Total kilocalorie/week | | Minutes high intensity exercise/week | | Total activity count per day/100000 | | Active minutes/day | |
| --- | --- | --- | --- | --- | --- | --- | --- | --- |
| n_participants_ | 690 | | 690 | | 492 | | 492 | |
| n_observations_ | 2882 | | 2887 | | 824 | | 824 | |
|  | β (se) | p | β (se) | p | β (se) | p | β (se) | p |
| *(Intercept)* | *6769 (690)* | *<.001* | *77.1 (35.4)* | *0.029* | *19.81 (0.74)* | *<.001* | *397.2 (13.57)* | *<.001* |
| Light | 957 (781) | 0.221 | 74.5 (41.2) | 0.070 | 0.53 (0.85) | 0.536 | 3.61 (15.76) | 0.819 |
| Moderate | **1737 (743)** | **0.020** | **153.0 (38.4)** | **<.001** | **2.28 (0.80)** | **0.005** | **33.38 (14.76)** | **0.024** |
| Intense | **3396 (863)** | **<.001** | **336.3 (44.0)** | **<.001** | **4.18 (0.96)** | **<.001** | **51.38 (17.43)** | **0.003** |
| Years prior to index | 201 (109) | 0.065 | 4.2 (6.3) | 0.506 | **0.77 (0.21)** | **<.001** | **14.28 (4.23)** | **0.001** |
| Light × Years prior | 52 (123) | 0.671 | -6.6 (7.1) | 0.357 | -0.40 (0.27) | 0.133 | -8.73 (5.39) | 0.106 |
| Moderate × Years prior | -39 (115) | 0.736 | -9.9 (6.6) | 0.134 | **-0.49 (0.23)** | **0.037** | **-9.26 (4.70)** | **0.049** |
| Intense × Years prior | -163 (126) | 0.197 | **-20.1 (7.3)** | **0.006** | **-0.71 (0.29)** | **0.014** | **-13.01 (5.82)** | **0.026** |

Supplementary Figure 1: Scatter plots of life course physical activity (LCPA) versus change over time in questionnaire based as well as accelerometer based physical activity measures for participants with at least two observations of the activity measure. Change over time for each participant was estimated as the individual slope from a linear mixed effects models with random intercept and random slope.


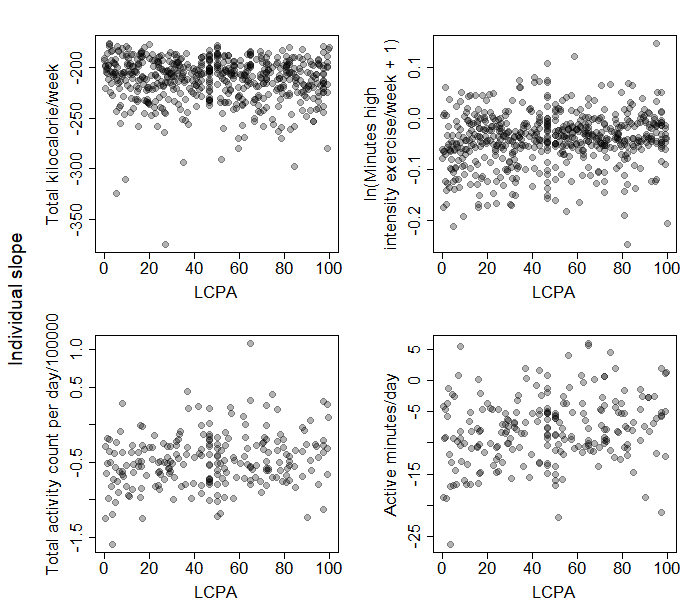


Supplementary Table 3: Coefficients from linear regression models estimating the association between life course physical activity and individual slopes from linear mixed effects models with random intercept and random slope for questionnaire based as well as accelerometer based physical activity estimated among participants with at least two observations of the activity measure.

| Individual slope | n | LCPA coefficient | |
| --- | --- | --- | --- |
|  |  | β (se) | p |
| Total kilocalorie/week | 575 | -0.061 (0.032) | 0.055 |
| ln(Minutes high intensity exercise/week + 1) | 575 | 0.0003 (0.0001) | <.001 |
| Total activity count per day/100000 | 227 | 0.003 (0.001) | 0.002 |
| Active minutes/day | 227 | 0.042 (0.012) | 0.001 |

Supplementary table 4: Coefficients estimating the association between scaled characteristics and scaled life course physical activity and scaled questionnaire based or accelerometer based physical activity slope from separate multivariable linear regression models adjusted for demographic, and technical covariates.

| Characteristic | n | LCPA | | Total kilocalorie/week | |
| --- | --- | --- | --- | --- | --- |
|  |  | β (se) p | 95% CI | β (se) p | 95% CI |
| HDL | 569 | 0.05 (0.04) 0.200 | -0.03, 0.13 | **0.12 (0.04) 0.005** | **0.04, 0.21** |
| Triglycerides | 569 | -0.07 (0.04) 0.103 | -0.14, 0.01 | -0.02 (0.05) 0.692 | -0.11, 0.07 |
| Hemoglobin | 567 | 0.03 (0.04) 0.425 | -0.04, 0.10 | -0.001 (0.04) 0.974 | -0.08, 0.08 |
| RDW | 569 | -0.04 (0.04) 0.336 | -0.12, 0.04 | -0.08 (0.05) 0.098 | -0.16, 0.01 |
| Peak VO_2_ | 473 | **0.22 (0.03) <.001** | **0.16, 0.29** | **0.08 (0.04) 0.035** | **0.01, 0.16** |
| FEV1/FVC | 502 | 0.03 (0.04) 0.552 | -0.06, 0.11 | 0.01 (0.05) 0.775 | -0.08, 0.11 |
| Fasting glucose | 550 | **-0.15 (0.04) 0.001** | **-0.24, -0.06** | -0.04 (0.05) 0.479 | -0.13, 0.06 |
| Fasting insulin | 550 | **-0.11 (0.05) 0.011** | **-0.20, -0.03** | -0.07 (0.05) 0.191 | -0.17, 0.03 |
| Thigh muscle area | 494 | **0.07 (0.02) 0.002** | **0.03, 0.12** | **-0.16 (0.03) <.001** | **-0.21, -0.11** |
| Thigh muscle intensity | 494 | **0.20 (0.04) <.001** | **0.13, 0.27** | **0.13 (0.04) 0.002** | **0.05, 0.21** |
| Abdominal subcutaneous fat | 478 | **-0.20 (0.05) <.001** | **-0.29, -0.11** | **-0.28 (0.05) <.001** | **-0.38, -0.18** |
| Visceral fat | 478 | **-0.08 (0.04) 0.031** | **-0.15, -0.01** | **-0.15 (0.04) <.001** | **-0.23, -0.07** |
| Usual gait speed | 570 | **0.17 (0.03) <.001** | **0.10, 0.23** | 0.04 (0.04) 0.331 | -0.04, 0.12 |
| Health ABC PPB | 566 | **0.25 (0.03) <.001** | **0.18, 0.31** | 0.04 (0.04) 0.345 | -0.04, 0.12 |
| Multimorbidity | 572 | **-0.22 (0.04) <.001** | **-0.30, -0.15** | -0.05 (0.04) 0.238 | -0.14, 0.03 |
|  |  |  |  |  |  |
| Characteristic | n | LCPA | | ln(Minutes high intensity exercise/week + 1) | |
|  |  | β (se) p | 95% CI | β (se) p | 95% CI |
| HDL | 569 | 0.05 (0.04) 0.200 | -0.03, 0.13 | 0.05 (0.04) 0.181 | -0.02, 0.13 |
| Triglycerides | 569 | -0.07 (0.04) 0.103 | -0.14, 0.01 | -0.03 (0.04) 0.393 | -0.12, 0.05 |
| Hemoglobin | 567 | 0.03 (0.04) 0.425 | -0.04, 0.10 | -0.03 (0.04) 0.414 | -0.10, 0.04 |
| RDW | 569 | -0.04 (0.04) 0.336 | -0.12, 0.04 | -0.02 (0.04) 0.637 | -0.10, 0.06 |
| Peak VO_2_ | 473 | **0.22 (0.03) <.001** | **0.16, 0.29** | **0.08 (0.03) 0.022** | **0.011, 0.14** |
| FEV1/FVC | 502 | 0.03 (0.04) 0.552 | -0.06, 0.11 | 0.03 (0.04) 0.501 | -0.06, 0.12 |
| Fasting glucose | 550 | **-0.15 (0.04) 0.001** | **-0.24, -0.06** | **-0.11 (0.05) 0.015** | **-0.20, -0.02** |
| Fasting insulin | 550 | **-0.11 (0.05) 0.011** | **-0.20, -0.03** | -0.05 (0.05) 0.290 | -0.14, 0.04 |
| Thigh muscle area | 494 | **0.07 (0.02) 0.002** | **0.03, 0.12** | 0.03 (0.02) 0.247 | -0.02, 0.07 |
| Thigh muscle intensity | 494 | **0.20 (0.04) <.001** | **0.13, 0.27** | **0.08 (0.04) 0.029** | **0.01, 0.15** |
| Abdominal subcutaneous fat | 478 | **-0.20 (0.05) <.001** | **-0.29, -0.11** | 0.001 (0.05) 0.990 | -0.09, 0.09 |
| Visceral fat | 478 | **-0.08 (0.04) 0.031** | **-0.15, -0.01** | -0.03 (0.04) 0.430 | -0.10, 0.04 |
| Usual gait speed | 570 | **0.17 (0.03) <.001** | **0.10, 0.23** | 0.03 (0.04) 0.332 | -0.04, 0.10 |
| Health ABC PPB | 566 | **0.25 (0.03) <.001** | **0.18, 0.31** | **0.09 (0.03) 0.007** | **0.03, 0.16** |
| Multimorbidity | 572 | **-0.22 (0.04) <.001** | **-0.30, -0.15** | -0.06 (0.04) 0.115 | -0.14, 0.01 |

Supplementary table 4: Continued.

| Characteristic | n | LCPA | | Total activity count per day/100000 | |
| --- | --- | --- | --- | --- | --- |
|  |  | β (se) p | 95% CI | β (se) p | 95% CI |
| HDL | 226 | 0.06 (0.06) 0.352 | -0.06, 0.18 | -0.02 (0.06) 0.731 | -0.14, 0.09 |
| Triglycerides | 226 | -0.06 (0.06) 0.340 | -0.19, 0.06 | 0.01 (0.06) 0.838 | -0.11, 0.13 |
| Hemoglobin | 225 | 0.04 (0.06) 0.525 | -0.08, 0.16 | 0.07 (0.06) 0.230 | -0.04, 0.18 |
| RDW | 225 | -0.02 (0.07) 0.739 | -0.16, 0.12 | 0.09 (0.07) 0.186 | -0.04, 0.22 |
| Peak VO_2_ | 185 | **0.14 (0.05) 0.004** | **0.05, 0.24** | 0.04 (0.05) 0.347 | -0.05, 0.13 |
| FEV1/FVC | 195 | -0.05 (0.06) 0.426 | -0.17, 0.07 | -0.04 (0.06) 0.501 | -0.16, 0.08 |
| Fasting glucose | 218 | -0.09 (0.08) 0.228 | -0.24, 0.06 | -0.08 (0.07) 0.291 | -0.22, 0.07 |
| Fasting insulin | 217 | **-0.16 (0.08) 0.045** | **-0.31, -0.004** | -0.05 (0.07) 0.490 | -0.20, 0.09 |
| Thigh muscle area | 196 | **0.10 (0.04) 0.008** | **0.03, 0.17** | 0.02 (0.03) 0.634 | -0.05, 0.08 |
| Thigh muscle intensity | 196 | **0.27 (0.06) <.001** | **0.16, 0.38** | 0.11 (0.05) 0.050 | 0.001, 0.22 |
| Abdominal subcutaneous fat | 189 | **-0.19 (0.06) 0.002** | **-0.30, -0.07** | **-0.14 (0.06) 0.010** | **-0.25, -0.04** |
| Visceral fat | 189 | -0.09 (0.06) 0.160 | -0.21, 0.03 | -0.01 (0.06) 0.873 | -0.13, 0.11 |
| Usual gait speed | 226 | **0.16 (0.06) 0.006** | **0.05, 0.27** | 0.05 (0.06) 0.329 | -0.05, 0.16 |
| Health ABC PPB | 226 | **0.30 (0.06) <.001** | **0.19, 0.41** | -0.01 (0.06) 0.866 | -0.12, 0.10 |
| Multimorbidity | 227 | **-0.16 (0.06) 0.010** | **-0.29, -0.04** | -0.004 (0.06) 0.942 | -0.12, 0.11 |
|  |  |  |  |  |  |
| Characteristic | n | LCPA | | Active minutes/day | |
|  |  | β (se) p | 95% CI | β (se) p | 95% CI |
| HDL | 226 | 0.06 (0.06) 0.352 | -0.06, 0.18 | 0.01 (0.06) 0.912 | -0.11, 0.12 |
| Triglycerides | 226 | -0.06 (0.06) 0.340 | -0.19, 0.06 | -0.02 (0.06) 0.700 | -0.14, 0.10 |
| Hemoglobin | 225 | 0.04 (0.06) 0.525 | -0.08, 0.16 | 0.08 (0.06) 0.142 | -0.03, 0.20 |
| RDW | 225 | -0.02 (0.07) 0.739 | -0.16, 0.12 | 0.07 (0.07) 0.301 | -0.06, 0.20 |
| Peak VO_2_ | 185 | **0.14 (0.05) 0.004** | **0.05, 0.24** | 0.06 (0.05) 0.188 | -0.03, 0.15 |
| FEV1/FVC | 195 | -0.05 (0.06) 0.426 | -0.17, 0.07 | 0.01 (0.06) 0.844 | -0.10, 0.13 |
| Fasting glucose | 218 | -0.09 (0.08) 0.228 | -0.24, 0.06 | -0.04 (0.07) 0.563 | -0.19, 0.10 |
| Fasting insulin | 217 | **-0.16 (0.08) 0.045** | **-0.31, -0.004** | -0.03 (0.07) 0.709 | -0.17, 0.12 |
| Thigh muscle area | 196 | **0.10 (0.04) 0.008** | **0.03, 0.17** | 0.02 (0.04) 0.505 | -0.05, 0.09 |
| Thigh muscle intensity | 196 | **0.27 (0.06) <.001** | **0.16, 0.38** | **0.13 (0.06) 0.024** | **0.02, 0.23** |
| Abdominal subcutaneous fat | 189 | **-0.19 (0.06) 0.002** | **-0.30, -0.07** | **-0.14 (0.06) 0.013** | **-0.25, -0.03** |
| Visceral fat | 189 | -0.09 (0.06) 0.160 | -0.21, 0.03 | -0.01 (0.06) 0.814 | -0.13, 0.10 |
| Usual gait speed | 226 | **0.16 (0.06) 0.006** | **0.05, 0.27** | 0.06 (0.06) 0.268 | -0.05, 0.17 |
| Health ABC PPB | 226 | **0.30 (0.06) <.001** | **0.19, 0.41** | 0.03 (0.06) 0.661 | -0.09, 0.14 |
| Multimorbidity | 227 | **-0.16 (0.06) 0.010** | **-0.29, -0.04** | 0.02 (0.06) 0.788 | -0.10, 0.14 |

Supplementary table 5: Coefficients for standardized LCPA from multivariable linear regression models with quantitative characteristics as the dependent variables (as measured or standardized) adjusted for questionnaire based or accelerometer based physical activity as well as demographic and technical covariates.

| Characteristic | n | As measured | | Standardized | | | |
| --- | --- | --- | --- | --- | --- | --- | --- |
|  |  | LCPA | | LCPA | | Total kilocalorie/week | |
|  |  | β (se) p | 95% CI | β (se) p | 95% CI | β (se) p | 95% CI |
| HDL, mg/dL | 680 | 1.02 (0.65) 0.119 | -0.26, 2.29 | 0.06 (0.04) 0.119 | -0.01, 0.13 | -0.06 (0.04) 0.151 | -0.14, 0.02 |
| Triglycerides, mg/dL | 680 | **-3.71 (1.82) 0.042** | **-7.28, -0.14** | **-0.08 (0.04) 0.042** | **-0.15, -0.003** | 0.03 (0.04) 0.458 | -0.05, 0.12 |
| Hemoglobin, g/dL | 680 | 0.03 (0.04) 0.470 | -0.05, 0.11 | 0.02 (0.03) 0.470 | -0.04, 0.09 | 0.02 (0.04) 0.513 | -0.05, 0.10 |
| RDW, % | 683 | **-0.07 (0.03) 0.047** | **-0.13, 0.00** | **-0.07 (0.04) 0.047** | **-0.14, -0.001** | -0.01 (0.04) 0.886 | -0.08, 0.07 |
| Peak VO_2_, ml/(kg × min) | 571 | **1.78 (0.24) <.001** | **1.31, 2.25** | **0.24 (0.03) <.001** | **0.18, 0.30** | -0.02 (0.03) 0.485 | -0.09, 0.04 |
| FEV1/FVC | 606 | 0.001 (0.003) 0.774 | -0.01, 0.01 | 0.01 (0.04) 0.774 | -0.07, 0.09 | 0.07 (0.04) 0.082 | -0.01, 0.16 |
| Fasting glucose, mg/dL | 655 | **-1.92 (0.60) 0.001** | **-3.10, -0.75** | **-0.13 (0.04) 0.001** | **-0.20, -0.05** | -0.05 (0.04) 0.259 | -0.13, 0.04 |
| Fasting insulin, mIU/mL | 655 | **-1.00 (0.35) 0.005** | **-1.68, -0.31** | **-0.11 (0.04) 0.005** | **-0.19, -0.03** | 0.07 (0.04) 0.113 | -0.02, 0.15 |
| Thigh muscle area, mm^2^ | 596 | **169 (67) 0.012** | **38, 300** | **0.05 (0.02) 0.012** | **0.01, 0.10** | **0.14 (0.02) <.001** | **0.10, 0.19** |
| Thigh muscle intensity, HU | 596 | **0.84 (0.14) <.001** | **0.56, 1.12** | **0.19 (0.03) <.001** | **0.13, 0.25** | 0.02 (0.03) 0.500 | -0.04, 0.09 |
| Abdominal subcutaneous fat, mm^2^ | 578 | **-1701 (319) <.001** | **-2326, -1076** | **-0.22 (0.04) <.001** | **-0.30, -0.14** | **0.14 (0.04) 0.002** | **0.05, 0.22** |
| Visceral fat, mm^2^ | 578 | **-774 (256) 0.003** | **-1276, -271** | **-0.10 (0.03) 0.003** | **-0.17, -0.04** | **0.08 (0.04) 0.045** | **0.002, 0.15** |
| Usual gait speed, m/s | 684 | **0.04 (0.01) <.001** | **0.02, 0.05** | **0.15 (0.03) <.001** | **0.09, 0.22** | **0.08 (0.04) 0.023** | **0.01, 0.15** |
| Health ABC PPB | 673 | **0.14 (0.02) <.001** | **0.10, 0.17** | **0.23 (0.03) <.001** | **0.17, 0.29** | **0.08 (0.03) 0.014** | **0.02, 0.15** |
| Multimorbidity | 685 | **-0.30 (0.05) <.001** | **-0.39, -0.20** | **-0.21 (0.03) <.001** | **-0.28, -0.14** | -0.003 (0.04) 0.931 | -0.08, 0.07 |

Supplementary table 5: Continued.

| Characteristic | n | As measured | | Standardized | | | |
| --- | --- | --- | --- | --- | --- | --- | --- |
|  |  | LCPA | | LCPA | | Minutes high intensity exercise/week | |
|  |  | β (se) p | 95% CI | β (se) p | 95% CI | β (se) p | 95% CI |
| HDL, mg/dL | 680 | 0.64 (0.66) 0.333 | -0.65, 1.93 | 0.04 (0.04) 0.333 | -0.04, 0.11 | **0.08 (0.04) 0.028** | **0.01, 0.15** |
| Triglycerides, mg/dL | 680 | -3.11 (1.85) 0.092 | -6.73, 0.50 | -0.07 (0.04) 0.092 | -0.14, 0.01 | -0.05 (0.04) 0.203 | -0.13, 0.03 |
| Hemoglobin, g/dL | 680 | 0.05 (0.04) 0.279 | -0.04, 0.13 | 0.04 (0.03) 0.279 | -0.03, 0.10 | -0.05 (0.03) 0.117 | -0.12, 0.01 |
| RDW, % | 683 | -0.06 (0.03) 0.063 | -0.13, 0.00 | -0.07 (0.04) 0.063 | -0.14, 0.004 | -0.02 (0.04) 0.546 | -0.09, 0.05 |
| Peak VO_2_, ml/(kg × min) | 571 | **1.64 (0.24) <.001** | **1.17, 2.11** | **0.22 (0.03) <.001** | **0.16, 0.28** | **0.08 (0.03) 0.008** | **0.02, 0.14** |
| FEV1/FVC | 606 | 0.001 (0.003) 0.808 | -0.01, 0.01 | 0.01 (0.04) 0.808 | -0.07, 0.09 | 0.04 (0.04) 0.263 | -0.03, 0.12 |
| Fasting glucose, mg/dL | 655 | **-1.79 (0.61) 0.003** | **-2.98, -0.60** | **-0.12 (0.04) 0.003** | **-0.19, -0.04** | -0.07 (0.04) 0.084 | -0.14, 0.01 |
| Fasting insulin, mIU/mL | 655 | **-0.92 (0.36) 0.010** | **-1.62, -0.22** | **-0.10 (0.04) 0.010** | **-0.18, -0.02** | -0.01 (0.04) 0.810 | -0.09, 0.07 |
| Thigh muscle area, mm^2^ | 596 | **195 (70) 0.005** | **58, 332** | **0.06 (0.02) 0.005** | **0.02, 0.11** | 0.04 (0.02) 0.063 | -0.002, 0.08 |
| Thigh muscle intensity, HU | 596 | **0.76 (0.14) <.001** | **0.48, 1.04** | **0.17 (0.03) <.001** | **0.11, 0.24** | **0.10 (0.03) 0.001** | **0.04, 0.16** |
| Abdominal subcutaneous fat, mm^2^ | 578 | **-1414 (324) <.001** | **-2049, -779** | **-0.18 (0.04) <.001** | **-0.26, -0.10** | **-0.10 (0.04) 0.011** | **-0.18, -0.02** |
| Visceral fat, mm^2^ | 578 | **-604 (260) 0.020** | **-1113, -95** | **-0.08 (0.04) 0.020** | **-0.15, -0.01** | **-0.07 (0.03) 0.042** | **-0.14, -0.003** |
| Usual gait speed, m/s | 684 | **0.03 (0.01) <.001** | **0.02, 0.05** | **0.15 (0.03) <.001** | **0.08, 0.21** | **0.07 (0.03) 0.042** | **0.003, 0.13** |
| Health ABC PPB | 673 | **0.14 (0.02) <.001** | **0.10, 0.17** | **0.22 (0.03) <.001** | **0.16, 0.28** | 0.05 (0.03) 0.086 | -0.01, 0.11 |
| Multimorbidity | 685 | **-0.30 (0.05) <.001** | **-0.39, -0.20** | **-0.21 (0.03) <.001** | **-0.28, -0.14** | -0.0002 (0.03) 0.995 | -0.07, 0.07 |

Supplementary table 5: Continued.

| Characteristic | n | As measured | | Standardized | | | |
| --- | --- | --- | --- | --- | --- | --- | --- |
|  |  | LCPA | | LCPA | | Total activity count per day/100000 | |
|  |  | β (se) p | 95% CI | β (se) p | 95% CI | β (se) p | 95% CI |
| HDL, mg/dL | 402 | 0.19 (0.86) 0.823 | -1.50, 1.89 | 0.01 (0.05) 0.823 | -0.08, 0.10 | **0.14 (0.05) 0.010** | **0.03, 0.24** |
| Triglycerides, mg/dL | 402 | -2.84 (2.43) 0.244 | -7.61, 1.93 | -0.06 (0.05) 0.244 | -0.16, 0.04 | -0.10 (0.06) 0.078 | -0.21, 0.01 |
| Hemoglobin, g/dL | 401 | **0.11 (0.05) 0.044** | **0.00, 0.22** | **0.09 (0.04) 0.044** | **0.003, 0.17** | -0.05 (0.05) 0.338 | -0.14, 0.05 |
| RDW, % | 402 | -0.05 (0.05) 0.312 | -0.14, 0.04 | -0.05 (0.05) 0.312 | -0.15, 0.05 | 0.02 (0.06) 0.669 | -0.09, 0.13 |
| Peak VO_2_, ml/(kg × min) | 347 | **1.66 (0.32) <.001** | **1.04, 2.28** | **0.22 (0.04) <.001** | **0.14, 0.30** | **0.13 (0.05) 0.004** | **0.04, 0.22** |
| FEV1/FVC | 380 | 0.002 (0.004) 0.630 | -0.01, 0.01 | 0.02 (0.05) 0.630 | -0.07, 0.12 | -0.02 (0.06) 0.700 | -0.13, 0.09 |
| Fasting glucose, mg/dL | 390 | **-1.87 (0.72) 0.010** | **-3.29, -0.46** | **-0.12 (0.05) 0.010** | **-0.21, -0.03** | -0.03 (0.05) 0.559 | -0.13, 0.07 |
| Fasting insulin, mIU/mL | 391 | **-1.34 (0.51) 0.008** | **-2.33, -0.35** | **-0.15 (0.06) 0.008** | **-0.26, -0.04** | -0.02 (0.06) 0.774 | -0.14, 0.10 |
| Thigh muscle area, mm^2^ | 357 | **282 (87) 0.001** | **111, 452** | **0.09 (0.03) 0.001** | **0.04, 0.15** | 0.02 (0.03) 0.446 | -0.04, 0.09 |
| Thigh muscle intensity, HU | 357 | **1.06 (0.19) <.001** | **0.70, 1.43** | **0.24 (0.04) <.001** | **0.16, 0.32** | **0.10 (0.05) 0.034** | **0.01, 0.19** |
| Abdominal subcutaneous fat, mm^2^ | 345 | **-1574 (444) <.001** | **-2444, -704** | **-0.20 (0.06) <.001** | **-0.31, -0.09** | **-0.15 (0.06) 0.020** | **-0.28, -0.03** |
| Visceral fat, mm^2^ | 345 | **-801 (357) 0.025** | **-1500, -102** | **-0.11 (0.05) 0.025** | **-0.20, -0.01** | -0.04 (0.06) 0.427 | -0.15, 0.06 |
| Usual gait speed, m/s | 403 | **0.03 (0.01) 0.003** | **0.01, 0.05** | **0.12 (0.04) 0.003** | **0.04, 0.21** | **0.14 (0.05) 0.004** | **0.04, 0.23** |
| Health ABC PPB | 400 | **0.13 (0.02) <.001** | **0.09, 0.18** | **0.22 (0.04) <.001** | **0.14, 0.29** | **0.12 (0.04) 0.007** | **0.03, 0.20** |
| Multimorbidity | 399 | **-0.24 (0.06) <.001** | **-0.37, -0.11** | **-0.17 (0.05) <.001** | **-0.26, -0.08** | -0.02 (0.05) 0.696 | -0.12, 0.08 |

Supplementary table 5: Continued.

| Characteristic | n | As measured | | Standardized | | | |
| --- | --- | --- | --- | --- | --- | --- | --- |
|  |  | LCPA | | LCPA | | Active minutes/day | |
|  |  | β (se) p | 95% CI | β (se) p | 95% CI | β (se) p | 95% CI |
| HDL, mg/dL | 402 | 0.30 (0.86) 0.725 | -1.38, 1.99 | 0.02 (0.05) 0.725 | -0.08, 0.11 | **0.13 (0.05) 0.012** | **0.03, 0.22** |
| Triglycerides, mg/dL | 402 | -3.06 (2.42) 0.207 | -7.80, 1.69 | -0.06 (0.05) 0.207 | -0.16, 0.04 | -0.09 (0.05) 0.090 | -0.20, 0.01 |
| Hemoglobin, g/dL | 401 | 0.10 (0.05) 0.058 | 0.00, 0.21 | 0.08 (0.04) 0.058 | -0.002, 0.16 | -0.01 (0.04) 0.837 | -0.10, 0.08 |
| RDW, % | 402 | -0.05 (0.05) 0.320 | -0.14, 0.05 | -0.05 (0.05) 0.320 | -0.15, 0.05 | 0.02 (0.05) 0.677 | -0.08, 0.12 |
| Peak VO_2_, ml/(kg × min) | 347 | **1.72 (0.32) <.001** | **1.10, 2.34** | **0.23 (0.04) <.001** | **0.15, 0.31** | 0.08 (0.04) 0.074 | -0.01, 0.16 |
| FEV1/FVC | 380 | 0.002 (0.004) 0.698 | -0.01, 0.01 | 0.02 (0.05) 0.698 | -0.08, 0.12 | 0.01 (0.05) 0.781 | -0.09, 0.12 |
| Fasting glucose, mg/dL | 390 | **-1.88 (0.72) 0.009** | **-3.28, -0.48** | **-0.12 (0.05) 0.009** | **-0.21, -0.03** | -0.04 (0.05) 0.432 | -0.14, 0.06 |
| Fasting insulin, mIU/mL | 391 | **-1.37 (0.50) 0.007** | **-2.35, -0.39** | **-0.15 (0.06) 0.007** | **-0.26, -0.04** | 0.01 (0.06) 0.895 | -0.11, 0.12 |
| Thigh muscle area, mm^2^ | 357 | **287 (87) 0.001** | **117, 456** | **0.09 (0.03) 0.001** | **0.04, 0.15** | 0.01 (0.03) 0.649 | -0.04, 0.07 |
| Thigh muscle intensity, HU | 357 | **1.09 (0.18) <.001** | **0.73, 1.45** | **0.25 (0.04) <.001** | **0.17, 0.33** | 0.08 (0.04) 0.061 | -0.003, 0.17 |
| Abdominal subcutaneous fat, mm^2^ | 345 | **-1637 (442) <.001** | **-2503, -770** | **-0.21 (0.06) <.001** | **-0.32, -0.10** | **-0.12 (0.06) 0.041** | **-0.24, -0.01** |
| Visceral fat, mm^2^ | 345 | **-823 (355) 0.021** | **-1519, -127** | **-0.11 (0.05) 0.021** | **-0.21, -0.02** | -0.03 (0.05) 0.592 | -0.13, 0.07 |
| Usual gait speed, m/s | 403 | **0.03 (0.01) 0.001** | **0.01, 0.05** | **0.14 (0.04) 0.001** | **0.05, 0.22** | 0.07 (0.04) 0.142 | -0.02, 0.15 |
| Health ABC PPB | 400 | **0.14 (0.02) <.001** | **0.09, 0.18** | **0.22 (0.04) <.001** | **0.15, 0.30** | **0.09 (0.04) 0.037** | **0.01, 0.16** |
| Multimorbidity | 399 | **-0.24 (0.06) <.001** | **-0.37, -0.12** | **-0.17 (0.05) <.001** | **-0.26, -0.08** | -0.001 (0.05) 0.984 | -0.09, 0.09 |

Supplementary text

Physical activity history questionnaire –


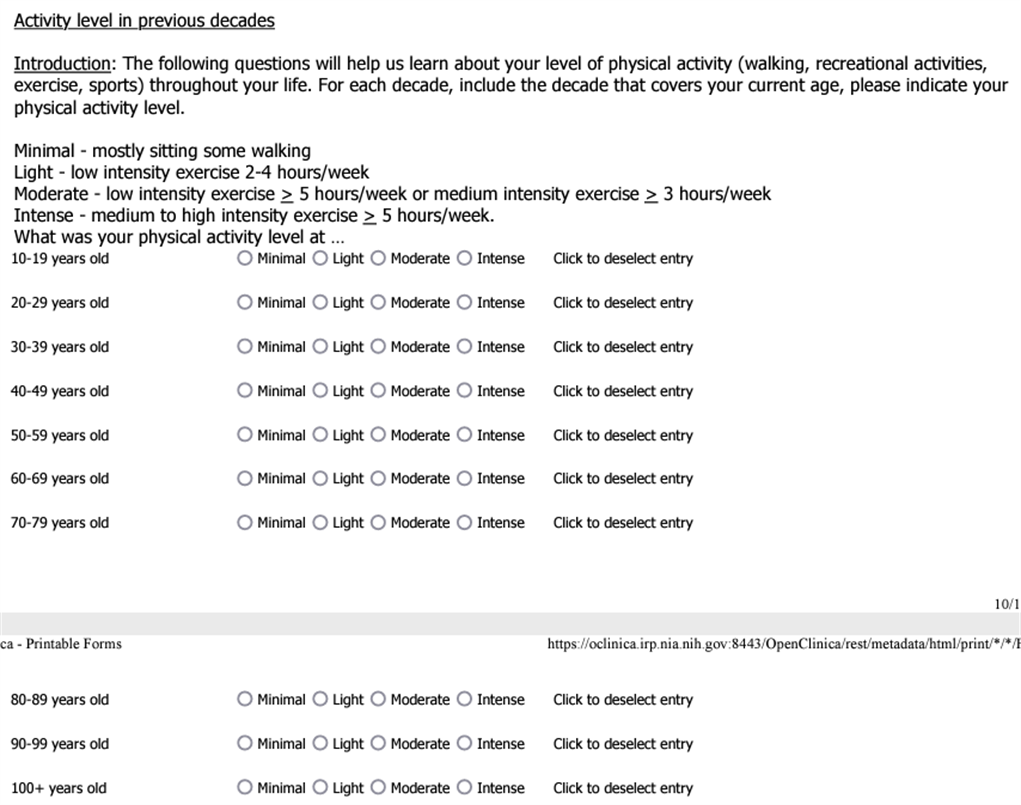


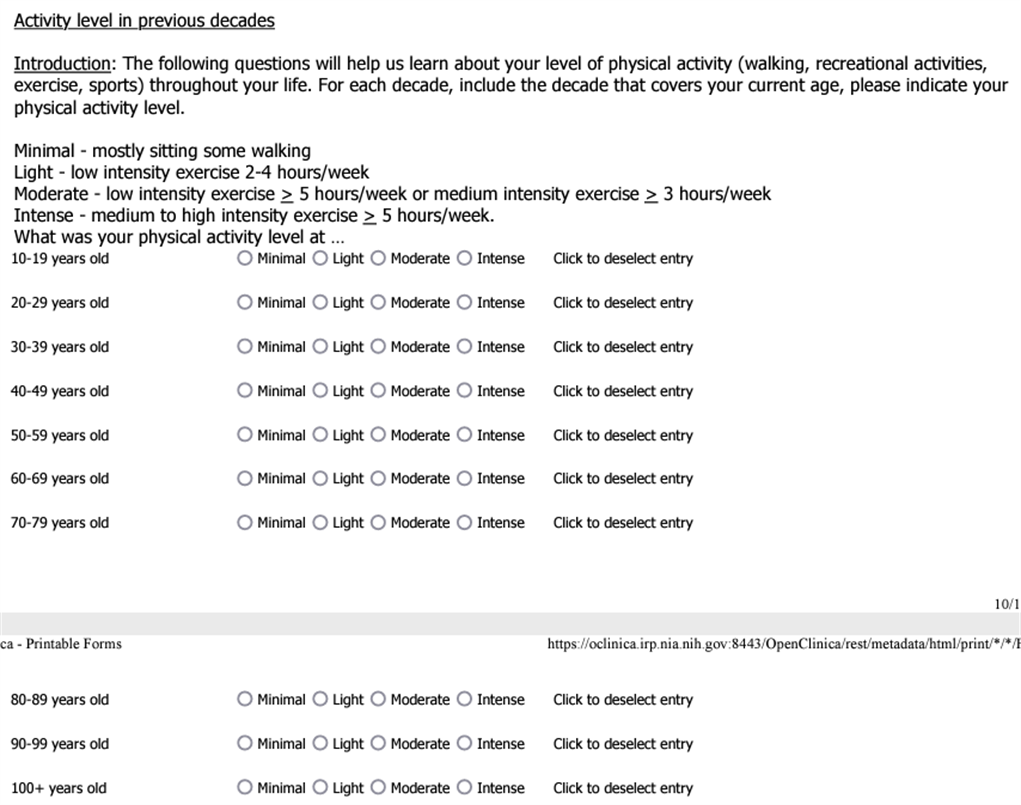

Supplement: Supplementary file 1 — Appendix S1: [file ACEL-23-e14078-s001.docx]
